# Congenital glossocervical fistula of presumed branchial origin: A unique anatomical pathway

**DOI:** 10.1016/j.bjorl.2025.101567

**Published:** 2025-03-24

**Authors:** Kento Koda, Kenji Kondo, Yuki Saito, Tatsuya Yamasoba

**Affiliations:** a University of Tokyo, Department of Otolaryngology and Head and Neck Surgery, Tokyo, Japan; b Tokyo Teishin Hospital, Department of Otolaryngology and Head and Neck Surgery, Chiyoda City, Japan

**Keywords:** External fistula, Computed tomography, Branchial region, Salivary gland fistula

## Introduction

Most congenital fistulas at the lateral neck occur because of developmental abnormalities of the second branchial apparatus and less frequently, the first branchial apparatus. These fistulas have characteristic anatomical pathways based on the embryological background. The second branchial fistula is formed from the tonsillar fossa to the anterior margin of the sternocleidomastoid muscle just above the clavicle.^1^ The first branchial fistula is formed on a line connecting the entrance to the external auditory canal to the hyoid bone.^1^ Here, we present a rare case of a glossocervical fistula with a unique internal travel pathway that differs from that of typical branchial fistulas.

## Case report

**Fig. 1:**
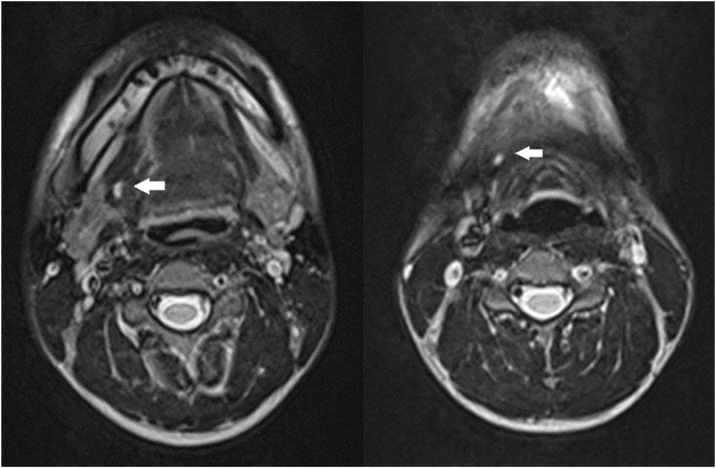
T2-weighted magnetic resonance imaging of the neck. The luminal structure extends from the surface of the right lateral anterior neck to the right oral floor.

A 17-year-old boy with no other medical history presented with mucus drainage from an opening on the right side of the neck during eating or drinking, which he experienced since childhood. Physical examination revealed a 2-mm-diameter orifice near the right lateral margin of the thyroid cartilage. Mucus discharge was observed from the orifice when the patient drank water. T2-weighted MRI of the neck revealed a luminal structure up to 5 mm in diameter that extended from the right anterior neck to the right oral floor through the anteromedial side to the right submandibular gland (Fig. 1).

**Fig. 2:**
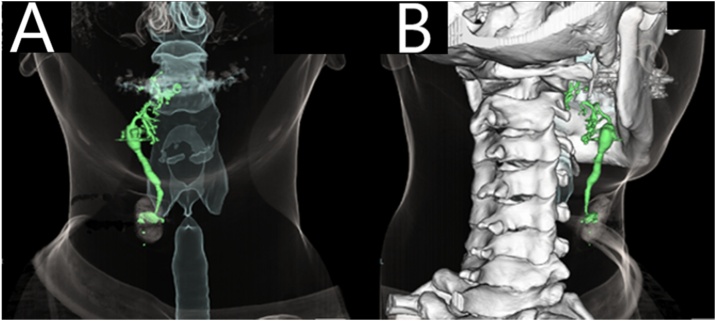
Cervical three-dimensional computed tomography fistulography. (A) The fistulous tract extends from the right lateral neck surface to the floor of the mouth. (B) A dendritic spread of contrast is observed at the root of the tongue.

To further assess the pathway of the fistulous tract, we performed Computed Tomography (CT) fistulography. We injected 1 mL of contrast agent (Iohexol 350 mgI/mL) through the cervical orifice using a 24-gauge catheter. Immediate three-dimensional CT showed that the tract was slightly dilated anterior to the submandibular gland and extended into the tongue. A dendritic spread of contrast was observed at the root of the tongue (Fig. 2).

**Fig. 3:**
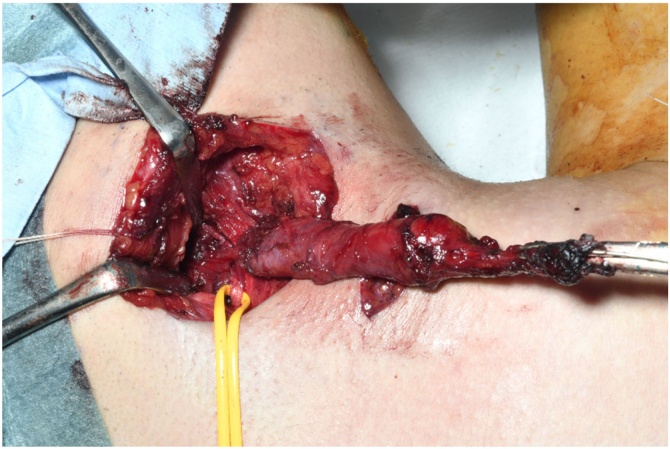
Cervical parallel skin incisions. An elliptical incision around the cervical orifice and a 7-cm transverse skin incision at the level of the hyoid bone are made. The fistulous duct is dissected upward from the surrounding tissue, appearing to taper and branch at the tongue root. A yellow vascular tape is applied over the hypoglossal nerve.

The tract was excised under general anaesthesia. Indigo carmine injection into the cannulated cervical orifice resulted in dye leakage from the tongue root. A parallel skin incision, an elliptical incision around the cervical orifice, and a 7-cm transverse skin incision at the level of the hyoid bone were made. The skin flap was elevated under the platysma muscle, and the fistulous duct was dissected upward from the surrounding tissue. The tract strayed under the anterior belly of the digastric muscle and extended through the anterior end of the submandibular gland and superficially to the hypoglossal and lingual nerves. At the tongue root, the tract, which was tapered and branched (Fig. 3), was ligated under the right oral floor mucosa between the sixth and seventh tooth from the midline and then excised. The submandibular gland and fistulous tract were not connected. Finally, the surgical field was thoroughly rinsed with saline solution, a drainage tube was placed, and the wound was sutured.

**Fig. 4:**
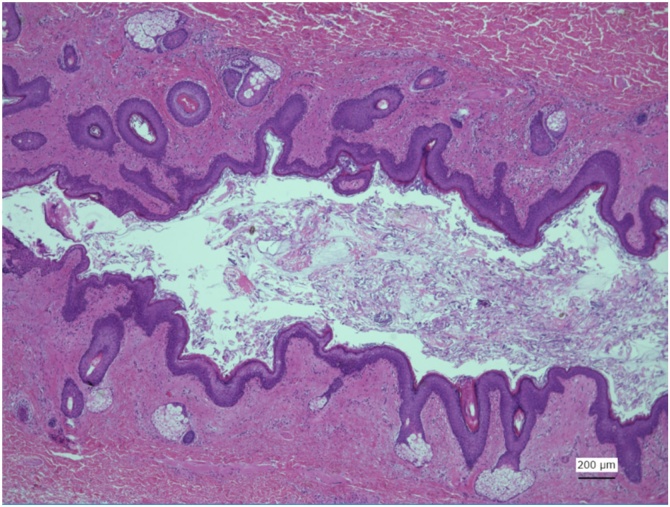
Micrograph of the section of the tract. The luminal surface is noted to be covered by squamous epithelium. Skin appendage-like structures, including sebaceous glands, sweat glands, and hair follicles, are observed. Salivary gland-like mixed mucous-plasma glands and conduits are also observed in the subepithelial areas.

Histological examination of the tract demonstrated that the luminal side was lined with keratinised stratified squamous epithelium with cutaneous adnexal structures such as sweat glands and hair follicles. Salivary glands and ducts were also observed along the duct (Fig. 4).

The 6-month postoperative period was uneventful with no apparent recurrence of symptoms such as mucus drainage or neck swelling.

## Discussion

The general location of the congenital fistula in our patient was similar to that of a second branchial fistula. Pathological examination revealed that the luminal side of the fistulous tract was lined with keratinised squamous epithelium, which implies that the fistula was of branchial origin. However, the anatomical pathway of the tract was different from that of a typical second branchial fistula, which commonly occurs at the anterior border of the sternocleidomastoid muscle, runs beneath the posterior belly of the digastric muscle, passes between the internal and external carotid arteries, and reaches the oropharynx — typically, the tonsillar fossa. However, the outer orifice of the fistula in this case was located more medially along the edge of the thyroid cartilage. It then ran upward beneath the anterior belly of the digastric muscle, passed anteriorly to the submandibular gland, superficially to the hypoglossal and lingual nerves, and finally reached the tongue root.

An alternative diagnosis of the fistula would be an aberrant salivary duct opening to the skin^2^ or a lateralised thyroglossal duct.^3^ However, in such lesions, the luminal lining of the duct is usually either non-keratinised squamous epithelium or ciliated epithelium, neither of which was observed in our case.

To the best of our knowledge, similar cases of congenital cervical fistulas with an anatomical pathway have rarely been reported.^4^, ^5^ Embryologically, the second pharyngeal arch overgrows the second, third, and fourth branchial clefts. This process results in the expansion of the second branchial clefts into an elongated common cavity called the sinus of His. If the sinus of His is not completely obliterated, various remnants originating from the second branchial cleft might develop. Such remnants may arise anywhere along the sinus of His. Thus, our case may represent a rare variation of the second branchial fistula with a unique anatomical pathway.

Our patient experienced mucus drainage while eating and drinking. Pathological examination of the fistulous duct revealed the presence of salivary glands in the duct. It has been reported that the branchial sinus could be connected to the salivary glands.^5^ Therefore, the surgery involved either complete excision of the fistulous tract and salivary glands or, if resection was not feasible, preservation of the internal drainage pathway to prevent cyst formation.

During surgery, indigo carmine injected through the external orifice was observed draining from the root of the tongue, confirming communication between the fistulous tract and the oropharynx. Due to the dendritic spread of the fistulae in the proximal region, identifying all of them was difficult. The fistulous duct was traced as far as possible, transected after ligation, and the proximal opening was left intact to prevent cyst formation from saliva accumulation.

The patient’s postoperative course was uneventful, with no signs of recurrence.

## Conclusion

In conclusion, we report a rare case of a glossocervical fistula with a unique anatomical pathway, different from that of typical branchial fistulas. Three-dimensional CT fistulography was useful for assessing the anatomical pathway of the fistulous tract. With this case, we are reminded that second branchial fistulas are highly variable.

## CRediT authorship contribution statement

Kento Koda: Investigation, Data curation, Writing – original draft.

Kenji Kondo: Conceptualization, Data curation, Writing – review & editing.

Yuki Saito: Software, Visualization.

Tatsuya Yamasoba: Project administration, Supervision.

## Consent to participate

This study was approved by the Institutional Review Board of the Graduate School of Medicine, University of Tokyo (protocol #2487). The patient and his parents provided informed consent for this study.

## Ethical standards

The authors assert that all procedures contributing to this work comply with the ethical standards of the relevant national and institutional guidelines on human experimentation (Graduate School of Medicine, University of Tokyo) and with the Helsinki Declaration of 1975, as revised in 2008.

## Funding

This research received no specific grant from any funding agency, commercial or not-for-profit sectors.

## Declaration of competing interest

The authors declare no conflicts of interest.
